# The presence of autoantibodies is associated with improved overall survival in lung cancer patients

**DOI:** 10.3389/fonc.2023.1234847

**Published:** 2023-09-20

**Authors:** Keying Jing, Huijuan Zhao, Jun Cai, Lianlian Chen, Peiming Zheng, Libo Ouyang, Gang Li, Rong Wang

**Affiliations:** ^1^ Henan University People's Hospital, Department of Clinical Laboratory, Henan Provincial People’s Hospital, Henan University, Zhengzhou, Henan, China; ^2^ Basic Medical College, Henan University of Science and Technology, Luoyang, Henan, China; ^3^ Henan Hospital of Integrated Chinese and Western Medicine, Zhengzhou, Henan, China

**Keywords:** autoantibody, antinuclear antibody, extractable nuclear antigen, lung cancer, overall survival

## Abstract

**Objective:**

Autoantibodies have been reported to be associated with cancers. As a biomarker, autoantibodies have been widely used in the early screening of lung cancer. However, the correlation between autoantibodies and the prognosis of lung cancer patients is poorly understood, especially in the Asian population. This retrospective study investigated the association between the presence of autoantibodies and outcomes in patients with lung cancer.

**Methods:**

A total of 264 patients diagnosed with lung cancer were tested for autoantibodies in Henan Provincial People’s Hospital from January 2017 to June 2022. The general clinical data of these patients were collected, and after screening out those who met the exclusion criteria, 151 patients were finally included in the study. The Cox proportional hazards model was used to analyze the effect of autoantibodies on the outcomes of patients with lung cancer. The Kaplan-Meier curve was used to analyze the relationship between autoantibodies and the overall survival of patients with lung cancer.

**Results:**

Compared to lung cancer patients without autoantibodies, those with autoantibodies had an associated reduced risk of death (HRs: 0.45, 95% CIs 0.27~0.77), independent of gender, age, smoking history, pathological type, and pathological stage of lung cancer. Additionally, the association was found to be more significant by subgroup analysis in male patients, younger patients, and patients with small cell lung cancer. Furthermore, lung cancer patients with autoantibodies had significantly longer survival time than those without autoantibodies.

**Conclusion:**

The presence of autoantibodies is an independent indicator of good prognosis in patients with lung cancer, providing a new biomarker for prognostic evaluation in patients with lung cancer.

## 1 Introduction

Lung cancer is the leading cause of cancer-related death in the world, and has the highest incidence and mortality of all malignant tumors in China (1, 2). Moreover, the recurrence rate of lung cancer remains high even after patients receive surgery, radiotherapy, chemotherapy (3), immunotherapy (4), gene therapy, and other treatments. Consequently, the disease remains a major public health concern worldwide. Recent surveys have shown that lung cancer mortality is still increasing in China (5, 6). To evaluate the prognosis of patients with lung cancer, clinicians can make judgements based on pathological type and pathological staging of lung cancer. However, the prognosis of patients with lung cancer is difficult to determine due to the heterogeneity that exists between different patients. It has been reported that the presence of autoantibodies is associated with the prognosis of cancers, such as colon cancer, breast cancer, and ovarian cancer (7–11).

Autoantibodies are antibodies produced by the body to target the constituents of its own tissue when immune tolerance is diminished. In systemic lupus erythematosus (SLE), the production of autoantibodies is abnormally increased, which becomes one of the important markers for the diagnosis and treatment of the disease. In the pathological process of SLE, autoantibodies promote inflammatory responses and tissue damage through a variety of mechanisms, which have an impact on the development and severity of the disease (12, 13).

Recent studies have found that the production of autoantibodies is not limited to autoimmune diseases, with their presence also having been detected in cancer patients (9). In fact, a previous study detected circulating antinuclear antibodies in the serum of 30% of cancer patients, whereas the same antibodies were absent or present at very low levels in normal human serum (14). In addition, autoantibodies are stable serological proteins, and although the corresponding antigen level is very low, they are present at high levels in the serum. As a biomarker, autoantibodies have been widely used in the early screening of a variety of cancers. Furthermore, autoantibodies are specific and detectable in different subtypes of lung cancer. Previous research showed that the EarlyCDT-Lung test could measure a highly specific biomarker of autoantibodies in the blood, assisting in the prediction of lung cancer risk (15, 16). The detection of autoantibodies can be performed by employing minimally invasive blood sampling techniques to obtain blood samples and by using a simple indirect immunofluorescence assay. A recent study showed that autoantibodies can be used as prognostic markers in patients with lung cancer, thereby providing a new basis for evaluating their survival status (17). However, the correlation between autoantibodies and the prognosis of lung cancer patients is not well known.

This study retrospectively analyzed the correlation between peripheral blood autoantibodies and the outcomes of lung cancer patients, providing a new method for monitoring the prognostic indicators of such patients.

## 2 Materials and methods

### 2.1 Study design and patients

This is a retrospective cohort study that included 264 patients who were diagnosed with lung cancer and underwent autoantibody testing at Henan Provincial People’s Hospital (Zhengzhou, China) from January 2017 to June 2022. The termination of the follow-up was June 2023. All patients with lung cancer were diagnosed according to the guidance of the National Comprehensive Cancer Network (18, 19). We excluded participants who had pre-existing autoimmune diseases (n = 5). Due to missing follow-up data (n = 108), 151 patients were finally included in the analysis.

The general clinical data of patients were collected from the hospital’s electronic medical records, including age, gender, smoking history, pathological type of lung cancer, pathological stage, and treatment. According to the Lung Cancer Screening, Version 3.2018 Clinical Practice Guidelines in Oncology, smoking history was defined as a patient who smoked continuously or cumulatively for 6 months or more over their lifetime (20). The types of lung cancer were classified by pathology, i.e., small cell lung cancer, adenocarcinoma, and squamous cell carcinoma. According to the 8th edition of the TNM classification of lung cancer as implemented by the Union for International Cancer Control, lung cancer patients were divided into four stages according to different TNM stages (21). Pathological stage was grouped into two subgroups, i.e., phase I & II, and phase III & IV, according to clinical value.

The outcome overall survival (OS) was defined as the time from diagnosis to death caused directly by the disease. Progression-free survival (PFS) was defined as the time from the diagnosis of disease to progression.

All methods were conducted in accordance with relevant guidelines and regulations. This study was approved by the Ethics Committee of Henan Provincial People’s Hospital (201950).

### 2.2 Laboratory indicator detection

The autoantibodies included autoantibodies for nuclear antigens (ANAs) and antibodies against extractable nuclear antigens (anti-ENAs) in this study.

ANAs were measured with the use of an indirect immunofluorescence assay (IFA) kit, following the manufacturer’s protocol (EUROIMMUN, Germany). ANAs were detected using HEp-2 cells and monkey liver biochip conjugated with specific anti-human IgG. The IFA on HEp-2 cells is the most frequently used method for screening for the presence of a vast array of autoantibodies and was considered the gold standard by the American College of Rheumatology (22). HEp-2 cells are human laryngeal carcinoma epithelioid cells with abundant nucleoplasm with a rich variety of abundant nuclear antigens, a large nucleus, and a clear cell structure making it easy to observe results and allowing fluorescent staining analysis. Monkey liver tissue is helpful to determine fluorescence patterns, especially in some undistinguishable patterns. The fluorescence characteristics in monkey liver tissue are key points of identification, thus avoiding mistakes (23, 24). In the first incubation step, specific antibodies from the diluted patient sample bind to the solid-phase bound antigens. In the next step, a fluorescein (FITC)-labelled antibody (conjugate) binds to the specific antibodies from the patient sample. By excitation with the respective wavelength, the complex can be made visible at the fluorescence microscope. Serum titers were measured from 1:100 to the end point, and the results were expressed as the last positive dilution. The positive quantitative results were 1:100, 1:320, 1:1000, 1:3200, etc. An ANAs titer ≥ 1:100 was considered a positive result.

The ANA patterns were recently defined by the International Consensus on ANA Patterns. In this study, the patterns of ANAs included nuclear speckled (27 cases), cytoplasmic speckled (14 cases), nucleolar (6 cases), nuclear dots (2 cases), rods and rings (1 case), nuclear homogeneous (1 case), centromere (1 case), Polar/Golgl-like (1 case), cytoplasmic fibrillar (1 case), and the combinations of patterns (8 cases).

Anti-ENAs were detected by the multi-parameter line immunoassay (LIA) using a EUROLineMaster automated immunoblot apparatus (EUROIMMUN, Germany). For detection membrane strips, the results were evaluated using the EUROLineScan software. The gray value was automatically identified by the analyzer (negative: ≤ 10; weakly positive: 11-25; strongly positive: ≥ 26). A gray value of 11 and greater was considered positive.

ENAs in the assay include 12 different antigens: nRNP, Sm, SS-A, Ro-52, SS-B, Scl-70, Jo-1, CENP B, dsDNA, nucleosomes, histones, and ribosomal P-protein. In our study, the positive anti-ENAs included anti-Ro-52 (12 cases), anti-SSA (3 cases), anti-CENP (2 cases), anti-dsDNA (2 cases), anti- nRNP (2 cases), anti- Scl-70 (1 case), anti- ribosomal P-protein (1 case), anti- Jo-1 (1 case), and the combinations (10 cases). Notably, both ANAs and anti-ENAs antibodies positivity can occur in one patient.

### 2.3 Statistical analysis

Continuous variables are shown as the mean ± standard deviation (SD), and categorical variables are shown as the frequency (%). Cox regression analysis was used to estimate the hazard ratios (HRs) and 95% confidence intervals (CIs) were used for the prognosis of lung cancer patients with different variables. Unadjusted and multivariable-adjusted models were used to assess the association between autoantibodies and outcomes in lung cancer patients. The crude model was an unadjusted model without adjustment for covariates. The adjusted I model was the least adjusted model with adjustments only for sex and age. The adjusted II model was adjusted for age, sex, smoking history, and the pathological type and pathological stage of the lung cancer as covariates. The adjusted III model was a model adjusted for age, sex, smoking history, the pathological type and stage of the lung cancer, and treatment. Subgroup analysis was performed using a stratified Cox regression model. Age was used as a continuous variable, and 60 years and older as the elderly (25), which were transformed into categorical variables, then the interaction test was conducted. Pathological type was classified into small cell lung cancer and non-small cell lung cancer according to clinical significance in the subgroup analysis. The OS curve was calculated by the Kaplan-Meier method and *P*<0.05 was considered statistically significant.

## 3 Results

### 3.1 Demographic and clinical characteristics of patients with lung cancer

Of the 151 patients enrolled, 83 patients survived and 68 died. The demographic and clinical characteristics of lung cancer patients are shown in **Table 1**. There were 111 males (73.5%) and 40 females (26.5%). The mean age of the patients was 61.9 years. 65 patients (43.0%) had no smoking history, and 86 patients (57.0%) had smoking history. 70 cases (46.4%) were autoantibody-positive and 81 cases (53.6%) were autoantibody-negative. As shown in **Table 1**, the lung cancer patients with autoantibodies had a significantly higher survival rate compare to the lung cancer patients without autoantibodies (*P* = 0.002). These results suggest that smoking history, pathological type, treatment, and the presence of autoantibodies are associated with the survival of patients with lung cancer.

**Table 1:** Demographic and clinical characteristics of the patients with lung cancer.

Variables	Total(n=151)	Survival (n=83)	Death (n=68)	*P* value
Sex, n (%)				0.063
Male	111 (73.5)	56 (67.5)	55 (80.9)	
Female	40 (26.5)	27 (32.5)	13 (19.1)	
Age, year, Mean ± SD	61.9 ± 9.9	61.2 ± 9.4	62.7 ± 10.5	0.348
Smoking history, n (%)				0.006
No	65 (43.0)	44 (53.0)	21 (30.9)	
Yes	86 (57.0)	39 (47.0)	47 (69.1)	
Pathological type, n (%)				< 0.001
Small cell lung cancer	61 (40.4)	21 (25.3)	40 (58.8)	
Adenocarcinoma of the lung	58 (38.4)	39 (47.0)	19 (27.9)	
Squamous cell carcinoma of lung	32 (21.2)	23 (27.7)	9 (13.2)	
Pathological stage, n (%)				0.207
Phase I & II	19 (12.6)	13 (15.7)	6 (8.8)	
Phase III & IV	132 (87.4)	70 (84.3)	62 (91.2)	
Treatment, n (%)				0.002
No treatment	6 (4.0)	2 (2.4)	4 (5.9)	
Surgical treatment	17 (11.3)	12 (14.5)	5 (7.4)	
Chemotherapy	63 (41.7)	24 (28.9)	39 (57.4)	
Targeted drug therapy	6 (4.0)	6 (7.2)	0 (0)	
Combination therapy	59 (39.1)	39 (47.0)	20 (29.4)	
Autoantibody, n (%)				0.002
Negative	81 (53.6)	35 (42.2)	46 (67.6)	
Positive	70 (46.4)	48 (57.8)	22 (32.4)	
ANAs titer, n (%)				0.053
Negative	89 (58.9)	41 (49.4)	48 (70.6)	
1:100	39 (25.8)	28 (33.7)	11 (16.2)	
1:320	17 (11.3)	10 (12)	7 (10.3)	
≥1:1000	6 (4.0)	4 (4.8)	2 (2.9)	
Anti-ENAs gray value, n (%)				0.395
Negative	117 (77.5)	62 (74.7)	55 (80.9)	
Weakly positive	12 (7.9)	6 (7.2)	6 (8.8)	
Strongly positive	22 (14.6)	15 (18.1)	7 (10.3)	
PFS, Mean ± SD	18.3 ± 17.6	23.0 ± 19.6	12.5 ± 12.6	< 0.001
OS, Mean ± SD	21.7 ± 17.9	25.9 ± 19.8	16.6 ± 13.8	0.001

ANAs, autoantibodies for nuclear antigens (IFA); Anti-ENAs, antibodies against extractable nuclear antigens (LIA); OS, overall survival; PFS, progression-free survival; SD, standard deviation.

Positive autoantibody was defined as positive ANAs and/or positive anti-ENAs. The gray value was automatically identified by the analyzer (negative: ≤ 10; weakly positive: 11-25; strongly positive: ≥ 26). P < 0.05 indicated a statistically significant difference.

Additionally, we divided 151 lung cancer patients into groups according to autoantibodies, and the results showed that there was significant difference between autoantibodies and the outcome of the patients with lung cancer (**Supplementary Table 1**).

### 3.2 Univariate cox regression models for lung cancer patients

**Table 2** shows the HRs and 95% CIs of different variables on the risk of the mortality outcome in patients with lung cancer. According to the results, compared to male patients, female patients had a 57% reduction in the risk of death (95% CIs 0.24, 0.79). Compared to lung cancer patients without smoking history, lung cancer patients with smoking history had a 1.43-fold increased risk of death (*P* < 0.001). Compared to patients with small cell lung cancer, patients with lung adenocarcinoma had a 62% (95% CIs: 0.22, 0.66) reduction in mortality, and patients with squamous cell cancer had a 59% (95% CIs: 0.2, 0.85) reduction in mortality. In addition, compared to the lung cancer patients without autoantibody, lung cancer patients with autoantibody had a 49% reduction in the risk of death (95% CIs: 0.3, 0.84).

**Table 2:** Univariate cox regression models for lung cancer patients.

Variables	HRs (95%CI)	*P* value
Sex, n (%)		
Male	Ref	
Female	0.43 (0.24,0.79)	0.007
Age, year, Mean ± SD	1.02 (0.99,1.04)	0.135
Smoking history, n (%)		
No	Ref	
Yes	2.43 (1.44,4.1)	< 0.001
Pathological type, n (%)		
Small cell lung cancer	Ref	
Adenocarcinoma of the lung	0.38 (0.22,0.66)	< 0.001
Squamous cell carcinoma of lung	0.41 (0.2,0.85)	0.016
Pathological stage, n (%)		
Phase I & II	Ref	
Phase III & IV	1.71 (0.74,3.95)	0.211
Treatment, n (%)		
No treatment	Ref	
Surgical treatment	0.32 (0.09,1.19)	0.09
Chemotherapy	1.02 (0.36,2.87)	0.973
Targeted drug therapy	0 (0, Inf)	0.996
Combination therapy	0.54 (0.18,1.58)	0.259
Autoantibody, n (%)		
Negative	Ref	
Positive	0.51 (0.3,0.84)	0.01
ANAs titer, n (%)		
Negative	Ref	
1:100	0.47 (0.24,0.91)	0.024
1:320	0.68 (0.31,1.51)	0.342
≥1:1000	1.35 (0.32,5.59)	0.681
Anti-ENAs gray value, n (%)		
Negative	Ref	
Weakly positive	0.9993 (0.4279,2.3341)	0.999
Strongly positive	0.75 (0.34,1.65)	0.473

ANAs, autoantibodies for nuclear antigens (IFA); Anti-ENAs, antibodies against extractable nuclear antigens (LIA); SD, standard deviation; Ref, reference; Inf, infinity.

Positive autoantibody was defined as positive ANAs and/or positive anti-ENAs. The gray value was automatically identified by the analyzer (negative: ≤ 10; weakly positive: 11-25; strongly positive: ≥ 26). Data presented are HRs and 95% CIs. P < 0.05 was considered statistically significant.

### 3.3 Multivariable cox regression models for lung cancer patients

Based on the previous results, ANAs titer and anti-ENAs gray value have no significant effect on the outcome of lung cancer patients. Therefore, we differentiated negatives and positives in subsequent analyses about ANAs and anti-ENAs. **Table 3** shows the HRs and 95% CIs for the risk of the mortality outcome by autoantibody presence. Taking the above results into consideration, multiple factors, such as gender, smoking history, pathological type and pathological stage, may affect the outcome of patients with lung cancer. In the unadjusted models, autoantibody-positive lung cancer patients had a 49% (95% CIs, 0.3, 0.86) lower risk of death compared to autoantibody-negative lung cancer patients. After adjusting for age and sex, the HRs were 0.51 (95% CIs: 0.3, 0.85, *P* for the trend = 0.009). The HRs were 0.53 (95% CIs: 0.32, 0.88, *P* for the trend = 0.014) after adjusting for age, sex, smoking history, and the pathological type and pathological stage of the lung cancer. Finally, after adjusting for age, sex, smoking history, the pathological type and stage of the lung cancer, and treatment, the HRs were 0.45 (95% CIs 0.27, 0.77; *P* for trend = 0.004), suggesting that the presence of autoantibodies is associated with improved outcomes in lung cancer patients.

**Table 3:** Multivariable cox regression models for lung cancer patients.

Variables	N. event (%)	Crude HRs (95% CIs)	Adjusted I HRs (95% CIs)	Adjusted II HRs (95% CIs)	Adjusted III HRs (95% CIs)
Autoantibody					
Negative	46 (56.8)	Ref	Ref	Ref	Ref
Positive	22 (31.4)	0.51 (0.31~0.86)	0.51 (0.3~0.85)	0.53 (0.32~0.88)	0.45 (0.27~0.77)
*P* value		0.01	0.009	0.014	0.004

Ref, reference.

Positive autoantibody was defined as positive ANAs and/or positive anti-ENAs. Data presented are HRs and 95% CIs. Adjusted model I adjusted for age and sex. Adjusted model II adjusted for age, sex, history of smoking, pathological type and pathological stage. Adjusted model III adjusted for age, sex, history of smoking, pathological, pathological stage and treatment. P < 0.05 was considered statistically significant.

### 3.4 Subgroup analyses

To further investigate whether the association between autoantibodies and outcomes in patients with lung cancer was stable across subgroups, analyses were performed stratified according to age, sex, smoking history, pathological type, pathological stage, and treatment (**Table 4**). Age was a continuous variable, with 60 years and older defined as the elderly, which was transformed into a categorical variable for subgroup analysis. Pathological type was classified into small cell lung cancer and non-small cell lung cancer according to clinical significance.

**Table 4:** Subgroup analyses.

Subgroup	Autoantibody		*P* value	*P* for interaction
	Negative	Positive		
Sex				0.533
Male	1(Ref)	0.47 (0.26~0.85)	0.013	
Female	1(Ref)	0.24 (0.05~1.13)	0.072	
Age				0.18
<60	1(Ref)	0.21 (0.07~0.62)	0.005	
≥60	1(Ref)	0.59 (0.31~1.13)	0.113	
Smoking history				0.173
No	1(Ref)	0.16 (0.05~0.52)	0.002	
Yes	1(Ref)	0.56 (0.3~1.05)	0.071	
Pathological type				0.947
Small cell lung cancer	1(Ref)	0.35 (0.17~0.71)	0.004	
Non-small cell lung cancer	1(Ref)	0.46 (0.19~1.1)	0.082	
Pathological stage, n (%)				0.353
Phase I & II	1(Ref)	0 (0~Inf)	0.995	
Phase III & IV	1(Ref)	0.5 (0.29~0.88)	0.016	
Treatment, n (%)				<0.001
No treatment	1(Ref)	4560425584948668416 (0~Inf)	0.999	
Surgical treatment	1(Ref)	0 (0~Inf)	0.999	
Chemotherapy	1(Ref)	0.28 (0.13~0.6)	0.001	
Targeted drug therapy	1(Ref)	1 (1~1)	N/A	
Combination therapy	1(Ref)	0.7 (0.28~1.79)	0.46	

Ref, reference; Inf, infinity; N/A, not applicable.

Positive autoantibody was defined as positive ANAs and/or positive anti-ENAs. Age is a continuous variable, which is converted into a categorical variable. Pathological type was classified into small cell lung cancer and non-small cell lung cancer according to clinical significance. P < 0.05 was considered statistically significant.

The data showed that, the correlation between autoantibodies and the outcome of the lung cancer patients was stable for gender (*P* for interaction = 0.533), age (*P* for interaction = 0.18), smoking history (*P* for interaction = 0.173), pathological type (*P* for interaction = 0.947) and pathological stage (*P* for interaction = 0.353), but not for different treatments (*P* for interaction < 0.001), suggesting that treatment plays an interactive role in the association. Furthermore, the associations were found to be more significant in female patients, younger patients, and patients with small cell lung cancer. Overall, the association between autoantibodies and the outcome of lung cancer was stable independent of sex, age, smoking history, pathological type, and pathological stage.

### 3.5 Survival analysis

**Figure 1:**
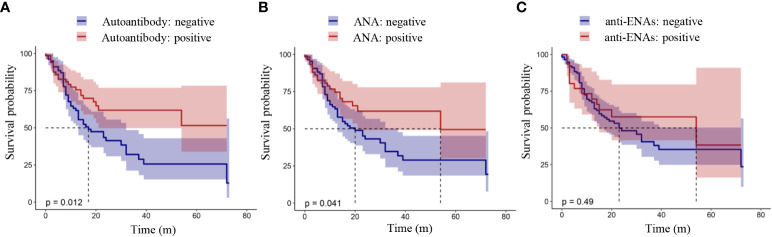
Progression-free survival analysis in patients with lung cancer. Positive autoantibody was defined as positive ANAs and/or positive anti-ENAs. ANAs, autoantibodies for nuclear antigens (IFA); anti-ENAs, antibodies against extractable nuclear antigens (LIA). *P* value < 0.05 indicated a statistically significant difference.

**Figure 2:**
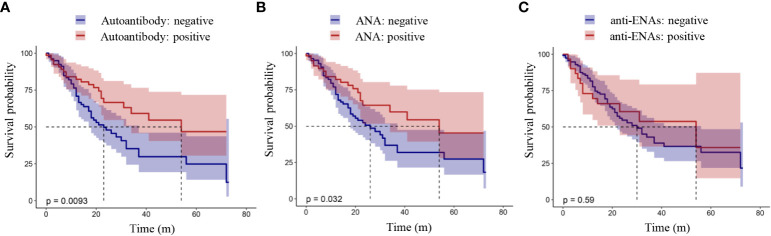
Overall survival analysis in patients with lung cancer. Positive autoantibody was defined as positive ANAs and/or positive anti-ENAs. ANAs, autoantibodies for nuclear antigens (IFA); anti-ENAs, antibodies against extractable nuclear antigens (LIA). *P* value < 0.05 indicated a statistically significant difference.

The relationship between the presence of autoantibodies and the OS or PFS of lung cancer patients was analyzed by a K-M curve. Shown in **Figure 1** is the relationship between the presence of autoantibodies and progression-free survival in lung cancer patients. The results showed that the PFS of lung cancer patients with autoantibodies was longer than that of patients without autoantibodies (*P* = 0.012, **Figure 1A**). Furthermore, lung cancer patients with ANAs had longer progression-free survival than those without ANAs (*P* = 0.041, **Figure 1B**). However, there was no significant difference between the survival of patients with anti-ENAs and those without anti-ENAs (*P* = 0.49, **Figure 1C**). Notably, we observed the similar results in the overall survival analysis (**Figure 2**). These results suggest that the presence of ANAs, rather than anti-ENAs, is associated with prolonged survival in lung cancer patients.

## 4 Discussion

In this retrospective cohort study, we found that the presence of autoantibodies was associated with improved OS in patients with lung cancer, independent of gender, age, smoking history, pathological type, and pathological stage of lung cancer.

The presence of antinuclear antibodies in tumor patients is not uncommonly reported (7–11, 26, 27). The loss of tolerance, inflammation, the changes in the expression levels of genes, protein structures and the changes of cell death mechanism may lead to the production of autoantibodies (28). However, loss of tolerance, inflammation, the changes in the expression levels of genes, protein structures and the changes of cell death mechanism affect the context in which the antigens are presented to the immune system, initiating the production of autoantibodies, in cooperation with other immune responses against transformed cancer cells. Due to the heterogeneity of cancer cells and the varied genetic and epigenetic differences between individual cancer patients, it is likely that anti-cancer humoral autoimmune responses originate from an array of such causes (29).

Anti-Ro52 is reportedly significantly increased in ovarian cancer patients and can be used as a marker to indicate better outcomes in such patients (11); antinuclear antibody positive breast cancer patients have prolonged survival after treatment, and the risk of disease recurrence and metastasis is low (10, 30); the presence of autoantibodies prolongs the progression-free survival of advanced non-small cell lung cancer patients (31) and so can be used as a prognostic factor for these patients (32). Consistent with these findings, our study found that the presence of autoantibodies was associated with longer overall survival in lung cancer patients. This may be due to the fact that the production of autoantibodies reflects a stronger immune response in cancer patients and therefore enhanced immune surveillance of cancer cells (26, 33, 34). This suggests that the immune system is more strongly activated in order to fight cancer cells, which promotes the occurrence of the autoimmune response.

However, there are also some contradictory results. With the presence of autoantibodies, there may occur opposite outcomes in different cancer types or in different stages of the same cancer. According to other studies, the presence of antinuclear antibodies may lead to poor outcomes in lung cancer patients treated with chemotherapy (3) and immunotherapy (4). The potential mechanism underlying this may be that antinuclear antibody can cause the body’s immune system to attack its own tissues and organs, which causes an inflammatory response and tissue damage, thus affecting the treatment effect and survival of such lung cancer patients (35); ANAs with a nucleolar pattern have been found significantly associated with reduced OS in patients with leukemia (36).

A certain correlation has been reported between autoantibodies and lung cancer in recent researches (8, 31, 32, 37). However, there are few studies on the correlation between autoantibodies and the outcomes of lung cancer in Asian population. A previous study showed that the presence of natural IgG antibodies in the body can be used as a prognostic indicator of non-small cell lung cancer (38). Our study reveals a correlation between the outcome of lung cancer patients and autoantibodies in Chinese population, providing a new means for monitoring the outcome of lung cancer patients in the Asian population.

There are certain limitations in our study. First of all, the sample size was small. Despite the large number of lung cancer patients admitted to our hospital, autoantibodies are considered to be screening indicators used to exclude autoimmune diseases, having limited use for cancer screening and outcome evaluation, thus resulting in the small number of enrolled patients in this retrospective study. Additionally, the study is a single-center study, which may cause potential bias. Secondly, the lung cancer patients in this study had three pathological types, and there existed great heterogeneity in the treatments and outcomes among patients of different pathological types. For example, the subgroup analysis revealed that different treatments played an interactive role in the association. One reason for this may be the limited number of patients who did not receive treatment and those who received targeted therapy, which led to potential bias. Furthermore, the detected autoantibodies in this study only included ANAs and anti-ENAs, while other autoantibodies, such as anti-thyroid antibodies, rheumatoid arthritis antibodies, auto-immune liver disease antibodies, were not included in this study. Interestingly, our results showed that ANAs, rather than anti-ENAs, were associated with the outcome of the patients with lung cancer, suggesting the number of lung cancer patients with certain antibody in anti-ENAs was too small to show a difference with the outcome of the patients. Additionally, the results could not be analyzed impartially due to the small sample size of certain pattern or antibody against certain antigen in the limited population of lung cancer patients with positive autoantibodies. Therefore, a larger sample size, stricter inclusion criteria, and the detection of a wider range of autoantibodies will be needed to increase the reliability of the study in the future.

In conclusion, the presence of autoantibodies was associated with improved overall survival in patients with lung cancer, independent of gender, age, smoking history, pathological type, and pathological stage of lung cancer in this retrospective cohort study, providing more options and methods for the monitoring of lung cancer outcomes. However, the understanding of the role of autoantibodies and their immune responses interact with the development of lung cancer remains unknown, and future research will be needed to explore its potential mechanism.

## Data availability statement

The raw data supporting the conclusions of this article will be made available by the authors, without undue reservation.

## Ethics statement

All methods were conducted in accordance with relevant guidelines and regulations. This study was approved by the Ethics Committee of Henan Provincial People’s Hospital (201950). The studies were conducted in accordance with the local legislation and institutional requirements. The participants provided their written informed consent to participate in this study. The manuscript presents research on animals that do not require ethical approval for their study.

## Author contributions

RW and GL conceived and designed the study. KJ and LC conducted the clinical data extraction and literature search. KJ, ZH, and LC conducted data analysis. KJ wrote the draft of the manuscript. HZ, JC, LC, PZ, LO, RW, and GL critically revised the manuscript. All authors approved submission of the final version of the manuscript.
