# Corrigendum: The presence of autoantibodies is associated with improved overall survival in lung cancer patients

**DOI:** 10.3389/fonc.2023.1353702

**Published:** 2023-12-19

**Authors:** Keying Jing, Huijuan Zhao, Jun Cai, Lianlian Chen, Peiming Zheng, Libo Ouyang, Gang Li, Rong Wang

**Affiliations:** ^1^ Henan University People's Hospital, Department of Clinical Laboratory, Henan Provincial People’s Hospital, Henan University, Zhengzhou, Henan, China; ^2^ Basic Medical College, Henan University of Science and Technology, Luoyang, Henan, China; ^3^ Henan Hospital of Integrated Chinese and Western Medicine, Zhengzhou, Henan, China

**Keywords:** autoantibody, antinuclear antibody, extractable nuclear antigen, lung cancer, overall survival

In the published article, there was an error in **affiliations 1**. Instead of **^1^Department of Clinical Laboratory, Henan Provincial People’s Hospital, People’s Hospital of Zhengzhou University, Henan University, Zhengzhou, Henan, China, **it should be **^1^Henan University People’s Hospital, Department of Clinical Laboratory, Henan Provincial People’s Hospital, Henan University, Zhengzhou, Henan, China.**

The authors apologize for this error and state that this does not change the scientific conclusions of the article in any way. The original article has been updated.

