# Supplementary material for: The presence of autoantibodies is associated with improved overall survival in lung cancer patients
Source: Front Oncol. 2023 Sep 20;13:1234847. doi: 10.3389/fonc.2023.1234847 (PMC10547871; doi:10.3389/fonc.2023.1234847)
Supplement: Supplementary file 1 [file Table_1.docx]

SUPPLEMENTARY TABLE 1 Demographic and clinical characteristics of the patients with lung cancer

| Variables | Total(n=151) | Autoantibody Negative (n=81) | Autoantibody Positive (n=70) | *P* value |
| --- | --- | --- | --- | --- |
| Sex, n (%) |  |  |  | 0.59 |
| Male | 111 (73.5) | 61 (75.3) | 50 (71.4) |  |
| Female | 40 (26.5) | 27 (32.5) | 13 (19.1) |  |
| Age, year, Mean ± SD | 61.9 ± 9.9 | 61.5 ± 10.4 | 62.3 ± 9.3 | 0.62 |
| Smoking history, n (%) |  |  |  | 0.538 |
| No | 65 (43.0) | 33 (40.7) | 32 (45.7) |  |
| Yes | 86 (57.0) | 48 (59.3) | 38 (54.3) |  |
| Pathological type, n (%) |  |  |  | 0.577 |
| Small cell lung cancer | 61 (40.4) | 35 (43.2) | 26 (37.1) |  |
| Adenocarcinoma of the lung | 58 (38.4) | 28 (34.6) | 30 (42.9) |  |
| Squamous cell carcinoma of lung | 32 (21.2) | 18 (22.2) | 14 (20.0) |  |
| Pathological stage, n (%) |  |  |  | 0.925 |
| Phase Ⅰ & Ⅱ | 19 (12.6) | 10 (12.3) | 9 (12.9) |  |
| Phase Ⅲ & Ⅳ | 132 (87.4) | 71 (87.7) | 61 (87.1) |  |
| Treatment, n (%) |  |  |  | 0.299 |
| No treatment | 6 (4.0) | 4 (4.9) | 2 (2.9) |  |
| Surgical treatment | 17 (11.3) | 12 (14.8) | 5 (7.1) |  |
| Chemotherapy | 63 (41.7) | 36 (44.4) | 27 (38.6) |  |
| Targeted drug therapy | 6 (4.0) | 3 (3.7) | 3 (4.3) |  |
| Combination therapy | 59 (39.1) | 26 (32.1) | 33 (47.1) |  |
| Outcome, n (%) |  |  |  | 0.002 |
| Survive | 83 (55.0) | 35 (43.2) | 48 (68.6) |  |
| Death | 68 (45.0) | 46 (56.8) | 22 (31.4) |  |
| PFS, Mean ± SD | 18.3 ± 17.6 | 17.9 ± 17.3 | 18.7 ± 17.9 | 0.767 |
| OS, Mean ± SD | 21.7 ± 17.9 | 21.1 ± 17.3 | 22.4 ± 18.7 | 0.656 |

**Abbreviations：**

OS, overall survival; PFS, progression-free survival; SD, standard deviation.

**Notes:**

Autoantibodies in this study included ANAs and/or anti-ENAs; Both ANAs and anti-ENAs positivity can occur in one patient. *P* < 0.05 indicated a statistically significant difference.
